# Thrombocytopenia in pregnant women with *Plasmodium falciparum* malaria in an area of unstable malaria transmission in eastern Sudan

**DOI:** 10.1186/1472-6890-12-10

**Published:** 2012-08-06

**Authors:** Mayyada B Adam, Gamal K Adam, Duria A Rayis, Mustafa I Elbashir, Ishag Adam

**Affiliations:** 1 Faculty of Medicine, University of Khartoum, P. O. Box 102, Khartoum, Sudan; 2 Faculty of Medicine, Gadarif University, P. O. Box 63, Gadarif, Sudan

## Abstract

**Background:**

Blood platelet levels are being evaluated as predictive and prognostic indicators of the severity of malaria infections in humans. However, there are few studies on platelets and *Plasmodium falciparum* malaria during pregnancy.

**Methods:**

A case–control study was conducted at Gadarif Hospital in Eastern Sudan, an area characterized by unstable malaria transmission. The aim of the study was to investigate thrombocytopenia in pregnant women with *P. falciparum* malaria (cases) and healthy pregnant women (controls).

**Results:**

The median (interquartile) platelet counts were significantly lower in patients with malaria (N = 60) than in the controls (N = 60), 61, 000 (43,000–85,000) vs. 249,000 (204,000–300,000)/μL, respectively, *p* < 0.001. However, there was no significant difference in the platelet counts in patients with severe *P. falciparum* malaria (N = 12) compared with those patients with uncomplicated *P. falciparum* malaria (N = 48), 68, 000 (33,000-88,000)/μL vs. 61, 000 (45,000**–**85,000)/μL, respectively, *p* = 0.8. While none of the control group had thrombocytopenia (platelet count <75, 000/μL), it was found that 6/12 (50%) and 27/48 (56.2%) (*p* <0.001) of the patients with severe malaria and uncomplicated malaria had thrombocytopenia, respectively. Pregnant women with *P. falciparum* malaria, compared with the pregnant healthy control group, were at higher risk (OR = 10.1, 95% CI = 4.1–25.18; *p* < 0.001) of thrombocytopenia. Two patients experienced bleeding, and there was one maternal death due to cerebral malaria where the patient’s platelet count was only 28,000/μL.

**Conclusion:**

*P. falciparum* malaria is associated with thrombocytopenia in pregnant women in this setting. More research is needed.

## Background

It has been estimated that, each year, 30.3 million African women become pregnant in malaria endemic areas [1]. Pregnant women are more susceptible to malaria infections than their non-pregnant counterparts [2]. Malaria during pregnancy is associated with poor maternal and fetal outcomes [3]. In Sudan, pregnant women in the eastern and central regions are susceptible to malaria infection regardless of their age or parity [4,5], and the disease is one of the leading causes of maternal and perinatal mortality [6-8].

Malaria, especially the severe *Plasmodium falciparum* form, can cause thrombocytopenia, where there is an abnormally low number of platelets, and activation of the coagulation cascade [9,10]. Hence, platelet count is being evaluated as a predictor and prognostic feature of malaria infection [11]. However, most research on thrombocytopenia and malaria is focused on non-pregnant populations including adults and children with symptomatic and asymptomatic *P. falciparum* infections [12-14]. There are few published data on platelets and *P. falciparum* infection during pregnancy [15]. The current study was conducted at Gadarif Hospital in Eastern Sudan to investigate thrombocytopenia in pregnant women infected with *P. falciparum* malaria parasites. The area is characterized by unstable malaria transmission; *P. falciparum* is the sole malaria species in the area [16], and the disease is a huge problem among pregnant women, regardless of their age or parity [17].

## Methods

A case–control study was conducted at the antenatal clinic in Gadarif Maternity Hospital in Eastern Sudan during the rainy/post rainy season of June-November 2011. Cases were pregnant women with *P. falciparum* malaria confirmed by blood film examination. Controls were afebrile healthy pregnant women attending for routine antenatal care without any illness, history, or symptoms (headache, dizziness, joint pain, anorexia, nausea, spontaneous bleeding) suggestive of malaria. In addition, the controls had parasite negative blood films. After signing an informed consent form, the obstetrics history was taken from cases and controls using pre-test questionnaires. Pregnant women with a hypertensive disorder of pregnancy (blood pressure of 140/90 mmHg or more, with or without proteinuria), or pregnant women using aspirin or heparin were not included in the case or control groups. The cases were examined thoroughly for signs of severe *P. falciparum* infection, and those with one or more criteria of severe malaria [18] were admitted to the hospital and managed accordingly, as we have recently described [19]. Women who had not fulfilled the criteria for severe infection were considered to have uncomplicated *P. falciparum* malaria and were treated with the first-line anti-malarial treatment (artesunate-sulfadoxine-pyrimethamine) as out-patients according to the Sudanese National Malaria control policy [20].

Blood films were prepared and stained with Giemsa, and 100× oil immersion fields were examined. All the slides were double-checked blindly and only considered negative if no parasites were detected in 100 fields. Densities (parasite per μL of whole blood) were then calculated on the basis of the WBC count of the individual subjects. An average of 500 WBCs were counted for each subject before parasite densities were estimated. Thin blood films were used for parasite species identification.

Two mL of blood were collected from each woman in the case and control groups. The blood was collected into ethylenediaminetetraacetic acid anticoagulant through venipuncture and kept on a roller with constant mixing. The complete blood count was determined using a Sysmex automated hematology analyzer in the lab within one hour of collection. Sysmex accuracy was determined on a daily basis using manufacturer-provided samples with known cell counts.

### Statistics

Data were entered in computer using SPSS (version 16.0 for Microsoft Windows) and double-checked before analysis. Continuous variables were checked for normality, and normally distributed data were described by the mean (standard deviation) and non-normally distributed data by the median (interquartile range). Categorical data were compared using a Chi-square test or a Fisher's exact test, as appropriate. Student's *t*-test and ANOVA were used to compare the means between two or three of the groups, respectively. Mann–Whitney and Kruskal-Wallis H tests were used to analyze medians. A Pearson correlation test was performed between the platelets and parasite counts. Univariate and multivariate analyses were used, with thrombocytopenia (platelet count <75, 000/μL) as the dependent variable, while age, parity, gestational age, and temperature were the independent factors. Odds ratios and 95% confidence interval were calculated, and a *P* value < 0.05 was considered significant.

### Ethics

The study received ethical clearance from the Research Board at the Faculty of Medicine, University of Khartoum, Sudan.

## Results

Sixty pregnant women presented with *P. falciparum* malaria during the study period, and an equal number of healthy pregnant women were assigned to the control group. Out of the 60 *P. falciparum*-infected women, 12 were diagnosed as severe *P. falciparum* cases that exhibited various clinical manifestations, including, severe anemia (3), hypotension (9), jaundice (3), cerebral malaria (1), hyperparasitemia (5), bleeding (2) and more than one criteria (7).

While there were no significant differences in the mean values (SD) for the age, parity, gestational age, temperature, or the biochemical tests between the severe malaria, uncomplicated malaria and the control groups, the hemoglobin values were significantly lower in patients with severe malaria (Table 1). The parasite count ranged from 5400 to 148350 ring-form parasites/μL with a geometric mean of 9225.7 rings/μL. None of these pregnant women had an enlarged spleen or liver. Two patients presented with nasal bleeding and bleeding of the gums.

**Table 1:** **Comparing mean(SD) of the basic clinical data between the patients with*P. falciparum*malaria and controls**

**Variables**	**Patients with severe malaria (*N*** **= 12)**	**Patients with uncomplicated malaria(*N*** **= 48)**	**Controls(*N*** **= 60)**	***P***
Age, years	26.7 (5.2)	26.0 (5.8)	25.8 (5.4)	0.8
Parity	3.0 (1.6)	3.2 (2.6)	2.4 (2.0)	0.1
Gestational age, weeks	24.7 (6.8)	25 (9.6)	25.8 (10.1)	0.8
Temperature, ^0^ C	38.5 (0.6)	37.7 (0.7)	37.1 (0.2)	0.3
Haemoglobin, g/dl	9.1 (2.2)	10.2 (1.6)	11.3 (1.3)	<0.001
Blood glucose, mg/dl	72.4 (3.0)	74.6 (4.0)	—	0.08
Serum bilirubin, mg/dl	2.0 (0.7)	1.8 (0.6)	—	0.3
Serum creatinine, mg/dl	1.0 (0.2)	1.0 (0.3)	—	0.9

While none of the controls had thrombocytopenia, it was found that 6/12 (50%) and 27/48 (56.2%), *p* < 0.001, of the patients with severe *P. falciparum* malaria and uncomplicated *P. falciparum* malaria had thrombocytopenia, respectively (Table 2).

**Table 2:** Comparison of n (%) of women with platelet concentration by categories x 1,000/μL between the cases and controls

**Platelets category**	**Patients with severe malaria (*N*** **= 12)**	**Patients with uncomplicated malaria (*N*** **= 48)**	**Controls (*N*** **= 60)**	***P***
<50	5 (41.7)	17 (35.4)	0 (0.0)	<0.001
50-74	1 (8.3)	10 (20.8)	0 (0.0)	0.002
75-99	5 (41.7)	15 (31.3)	1 (1.7)	<0.001
100-149	1 (8.3)	3 (6.3)	1 (1.7)	0.08
≥150	0 (0.0)	3 (6.3)	58 (96.7)	<0.001

**Figure 1:**
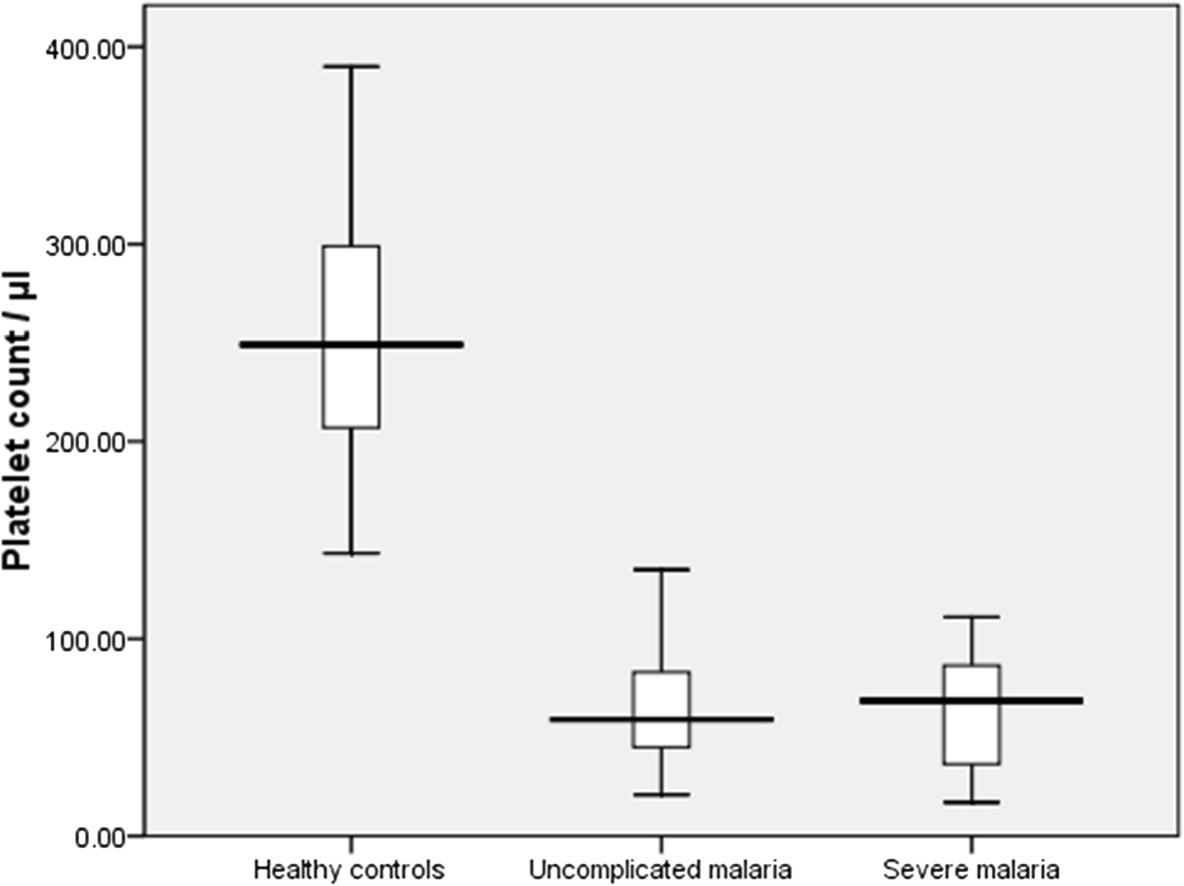
** Comparing platelets count between the patients with *P. falciparum *malaria and controls**.

In patients with *P. falciparum* malaria, the platelet count ranged from 17,000 to 311,000/μL. The median (interquartile range) of the platelets was significantly lower in patients with malaria (both severe and uncomplicated *P. falciparum* malaria) than in the controls, 61,000 (43,000–85,000) vs. 249,000 (204,000–300,000) (controls), *p* < 0.001. However, there was no significant difference in the median (interquartile range) of the platelets in patients with severe malaria compared with those with uncomplicated *P. falciparum* malaria, 68,000 (33,000–88,000) vs. 61,000 (45,000–85,000), respectively, *p* = 0.8, Figure 1.

The univariate and multivariate analyses found that malaria was the only risk factor for thrombocytopenia (odds ratio = 10.1, 95% CI = 4.1–25.18; *p* < 0.001) (Table 3). There was no significant correlation between the platelet levels and parasite counts (*r* = 0.124; P = 0.3). One patient died during resuscitation, after presenting with cerebral malaria and minor bleeding from the gums. This patient’s hemoglobin and platelet counts were 5 g/dl and 18, 000, respectively.

**Table 3:** Risk factors for thrombocytopenia using univariate and multivariate analyses

	**Univariate analysis**			**Multivariate analysis**		
**The variable**	**OR**	**95 % CI**	***P***	**OR**	**95 % CI**	***P***
Age	0.9	0.9–1.0	0.6	1.0	0.9–1.1	0.9
Primigravidae	0.9	0.8–1.1	0.6	0.8	0.6–1.0	0.3
Gestational age	1.0	0.9–1.0	0.9	1.0	0.9–1.0	0.6
Temperature	0.9	0.8–1.0	0.5	0.9	0.7–1.1	0.4
Haemoglobin	1.9	0.8–4.2	0.1	1.0	0.7–1.3	0.8
Malaria	8.5	3.6–20.8	<0.001	10.1	4.1–25.18	<0.001

## Discussion

To the best of our knowledge, this is the first published data on thrombocytopenia in pregnant women infected with *P. falciparum* malaria in Africa. The main findings of the current study were that the platelet counts in pregnant women with *P. falciparum* malaria were significantly lower than in the controls, and there was no difference in the platelet counts in women with severe *P. falciparum* malaria vs. in the women with uncomplicated malaria. One noteworthy finding was that pregnant women with malaria had a 10 times higher risk of thrombocytopenia than did the controls. In women with *P. falciparum* malaria infections, there was no significant correlation between parasite and platelet counts. Recently, Tan et al. observed that pregnant women living at the Thai-Burmese border were more susceptible to thrombocytopenia (platelet counts <150,000/μL) than their non-pregnant counterparts; the median platelet counts 134,000 (11,000–690,000) in this study were significantly lower in pregnant women with *P. falciparum* malaria and *Plasmodium vivax* malaria, with the first malaria episode increasing the risk of thrombocytopenia [15]. Interestingly, various definitions of platelet density (per μL) have been used to define thrombocytopenia in pregnant women, i.e. 75,000/μL, 115,000/μL and 150,000/μL, and of course, pregnancy itself can lead to thrombocytopenia [21-24]

In the current study, no correlation was found between the blood-stage parasite numbers and the platelet counts. This is in accordance with a previous observation, where it was shown that the pattern of thrombocytopenia and parasitemia did not have a linear relationship among malaria-infected Nigerian children [14]. However, among Papuan adults, platelet counts were negatively correlated with the level *P. falciparum* parasitemia [12]. Thrombocytopenia in malaria probably occurs through peripheral destruction, sequestration or excessive removal of the platelets by the spleen, as well as platelet consumption by the process of disseminated intravascular coagulation [25-28]. Platelets have been reported to enhance clumping of *P. falciparum*-infected erythrocytes, and this process might lead to pseudo thrombocytopenia [29]. In endemic areas, malaria patients have high levels of specific antibodies (IgG) which bind to platelet-bound malaria antigens [30].

In the current study, two patients presented with nasal bleeding and bleeding of the gums, and one of those who presented with cerebral malaria died. There were no episodes of bleeding or maternal death in our recent study on severe malaria among pregnant women in eastern Sudan [19]. Thrombocytopenia and spontaneous bleeding among pregnant women might cause life threatening antepartum or postpartum hemorrhages. Recent observations document that women with placental malaria are at higher risk of postpartum hemorrhage [31,32]. The question of malaria-associated hemorrhage in pregnancy might be addressed by longitudinal studies aimed at investigating the role played by platelets in peripheral and placental malaria.

In the current study, no significant difference was found in the platelet counts when patients with severe malaria were compared with those who had uncomplicated disease. This might be related to the small sample size in this study because only 12 patients presented with severe *P. falciparum* malaria. Nonetheless, Saravu et al. recently found that non-pregnant adult patients with severe *P. falciparum* malaria had a statistically significant lower platelet count than patients with uncomplicated malaria [33]. One of the limitations of the current study was the lack of the patients` outcomes in term of the treatment and the pregnancy outcomes.

## Conclusion

*P. falciparum* malaria is associated with thrombocytopenia in pregnant women in eastern Sudan. More research is needed.

## Competing interests

The authors declare that they have no competing interests.

## Authors’ contributions

MBA and IA carried out the study and participated in the statistical analysis. GKA and DAR participated in the clinical work and statistical analysis. MIE and DAR conducted the laboratory work. All the authors have read and approved the manuscript.

## Pre-publication history

The pre-publication history for this paper can be accessed here:

http://www.biomedcentral.com/1472-6890/12/10/prepub
